# Curcumin-Based Supplement for Vitreous Floaters Post-Nd:YAG Capsulotomy: A Pilot Study

**DOI:** 10.3390/vision9040098

**Published:** 2025-12-16

**Authors:** Alex Malandrini, Giovanni Rubegni, Davide Marini, Giulia Spadavecchia, Gian Marco Tosi

**Affiliations:** Ophthalmology Unit, Department of Medicine, Surgery and Neurosciences, University of Siena, 53100 Siena, Italy; alexmalandrini@gmail.com (A.M.); davide.marini@student.unisi.it (D.M.); giu.spada95@gmail.com (G.S.); gianmarco.tosi@unisi.it (G.M.T.)

**Keywords:** vitreous floaters, curcumin, bromelain, vitreous constituents, Nd:YAG laser

## Abstract

**Background**: To evaluate the short-term effects of a dietary supplement containing curcumin, bromelain, glucosamine, chondroitin sulphate, sodium hyaluronate, type II collagen, and vitamin C on symptomatic vitreous floaters (SVFs) following Nd:YAG laser capsulotomy. **Methods**: Forty eyes with SVFs on the first postoperative day were randomized into a control group (standard topical therapy, n = 20) and a treatment group (oral supplement plus standard therapy, n = 20). Outcomes included best-corrected visual acuity (BCVA), contrast sensitivity (CS), and subjective scores from a non-standardized questionnaire on floater perception (QS1), interference with daily activities (QS2), and foreign body sensation (QS3). Objective evaluation was performed using two novel ultrasound-based methods: mean number of vitreous peaks (MVP) from A-scans and mean grey intensity (MGI) from B-scan images processed with ImageJ. **Results**: At 2 months, the treatment group showed greater improvement in CS (Δ = 0.26 LogCS, CI, 0.14–0.38; *p* < 0.01), QS1 (Δ = 1.10; 95% CI, 0.60–1.60; *p* < 0.01), QS2 (Δ = 0.90; 95% CI, 0.40–1.40; *p* < 0.01), QS3 (Δ = 0.90; 95% CI, 0.44–1.36; *p* < 0.01), MVP (Δ = 1.10; 95% CI, 0.60–1.60; *p* < 0.01), and MGI (Δ = 12.89 units; 95% CI, 7.84–17.93; *p* < 0.01). BCVA was comparable between groups (*p* = 0.478). **Conclusions**: Short-term dietary supplementation with vitreous-specific nutrients is well tolerated and associated with improvements in reducing SVFs and foreign body sensations after Nd:YAG capsulotomy and may represent a promising non-invasive therapeutic option.

## 1. Introduction

Posterior capsular opacity (PCO) is a common postoperative complication of cataract surgery that can potentially reduce visual acuity and contrast sensitivity [1]. In the modern era, the 5-year incidence of PCO is up to 34% [2]. Posterior capsulotomy with a Q-switched Neodymium-doped crystal of Yttrium Aluminum Garnet (Nd:YAG) laser represents the standard treatment [3].

Symptomatic vitreous floaters (SVFs) or opacities are a possible side effect of Nd:YAG capsulotomy [4], with a 1-month incidence ranging between 20 and 50% depending on the technique employed [5,6]; they may be troublesome to patients [7], yet there is currently no established treatment for them [7].

The current treatment options for SVFs are quite limited [7] and, since available techniques are all invasive, most patients are managed conservatively with clinical observation [7,8], and reassured that they will adapt to the symptoms [7].

Surgical vitrectomy (floaterectomy) removes opacities definitively and is associated with a significant improvement of contrast sensitivity, glare, and questionnaire scores, but a marginal gain of visual acuity; however, adverse events include cataract in phakic patients, retinal detachment, cystoid macular edema, macular pucker, glaucoma or hypotony, endophthalmitis, and anesthesia-related risks [7].

Nd:YAG laser vitreolysis (floaterrhexis) reduces large floaters into smaller pieces. However, the efficacy has only been evaluated by a few small studies with non-standardized questionnaires and without objective measures, it requires multiple treatment sessions, and reported adverse events include cataract, retinal detachment, retinal hemorrhage, scotomata, worsening of floaters [9,10], uveitis, and ocular hypertension [7]. Pharmacological vitreolysis should enzymatically dissolve floaters; however, there is no approved molecule, and no clinical study has been conducted yet [7].

Hence, dietary intervention through oral supplements could represent the initial non-invasive approach for the management of SVFs, even following Nd:YAG capsulotomy.

Curcumin (diferuloylmethane) is the primary, yellow-coloured polyphenol in the rhizome of *Curcuma longa*, the Indian plant commonly known as turmeric [11], with anti-inflammatory and antioxidant properties attributed to the inhibition of NF-κB and various other intracellular factors and cytokines [12]. Due to its poor intestinal absorption owing to hydrophobicity, the formulation with piperine (alkaloid extract from black pepper, Piper nigrum) presents itself as an option to enhance systemic bioavailability by up to 2000% [13]. Curcumin has demonstrated several biological effects in vitro, in animal models, and in clinical studies of ocular diseases [14,15]. For instance, it promotes corneal wound healing and epithelial barrier protection, it has shown efficacy in treating dry eye (anti-inflammatory) [14,15], and it also offers benefits in the prevention of glaucoma (neuroprotective) [14,15], age-related macular degeneration (anti-apoptotic) [14], and diabetic retinopathy (antioxidant and anti-angiogenic) [14].

Bromelain is a fibrinolytic enzyme, originating from the stem of pineapples (*Ananas comosus* L. Merr.), exhibiting anti-inflammatory and anti-edematous properties, along with a recognized proteolytic function on primary and hematic vitreous floaters [16,17]. Type II collagen and mucopolysaccharides, glucosamine, chondroitin sulphate, and sodium hyaluronate, constitute the fibrillary structure of vitreous, are often associated with vitamin C, a known cofactor in collagen synthesis, and are thought to remodel and repair the fibrillary vitreous structure disrupted by the natural ageing process (synchysis and syneresis) [7].

The aim of this pilot study is indeed to evaluate, for the first time, the short-term efficacy of a commercially available oral supplement containing a mixture of curcumin, bromelain, and vitreous constituents in reducing SVFs induced by Nd:YAG laser capsulotomy, in terms of visual acuity, contrast sensitivity, ultrasonography quantification, and subjective perception of floaters.

## 2. Materials and Methods

**Figure 1:**
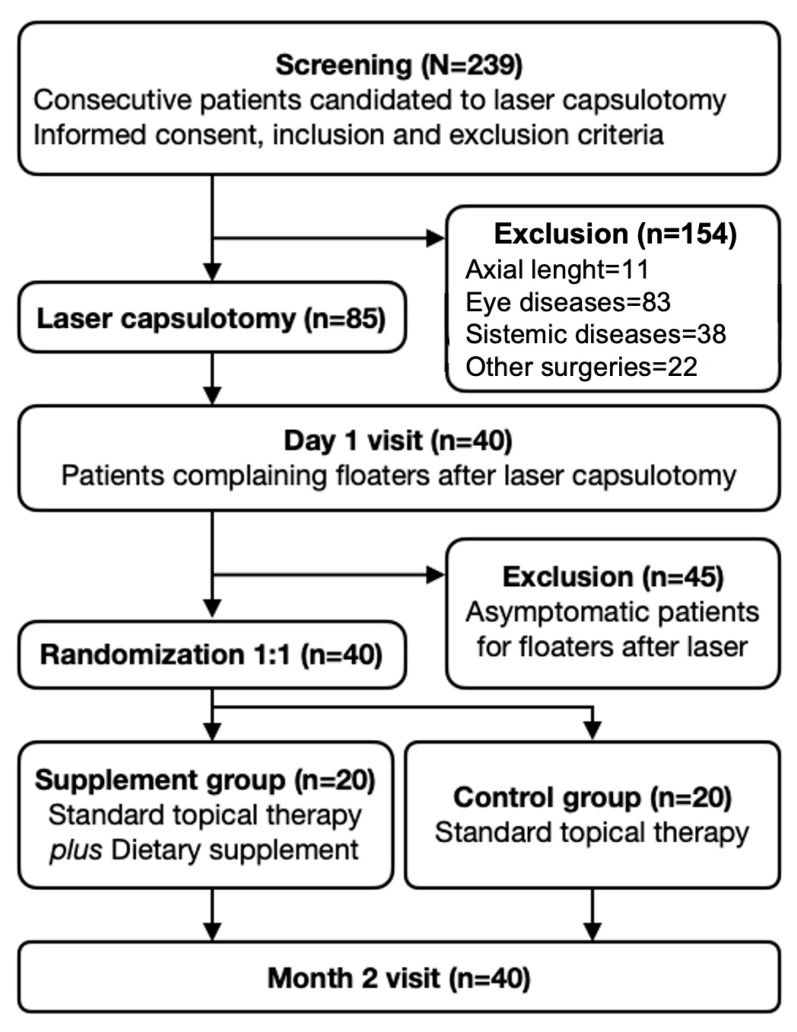
Study protocol and timeline.

This research was a pilot study in which participants were divided in two groups, both following the standard eyedrop therapy after laser capsulotomy, one with a commercially available dietary supplement (dietary intervention group) and the other without (control group, Figure 1). The primary outcome was contrast sensitivity (CS), given its relevance to the functional impact of symptomatic vitreous floaters. Secondary outcomes included the subjective improvement of floater symptoms (as assessed through a patient-reported questionnaire), the objective reduction in vitreous opacities (measured with ultrasound), and best-corrected visual acuity (BCVA).

This study was conducted in accordance with the principles of the Declaration of Helsinki and received conditional approval from the Territorial Ethics Committee of Toscana Region (Comitato Etico Regionale per la Sperimentazione Clinica della Regione Toscana, Siena, Italy; Opinion No. CET 299665). Informed consent was obtained from all participants after detailed explanation of the nature, purpose, and design of the study.

All consecutive patients referred to the laser service of the Ophthalmology Unit of the Department of Medicine, Surgery and Neuroscience, Siena University Hospital (Siena, Italy), from January 2022 to February 2023, were screened for eligibility.

Inclusion criteria comprised the following: phacoemulsification surgery with monofocal intraocular lens (IOL) implantation, PCO after uneventful cataract surgery, PCO-related clinically significant reduction in distance best-corrected visual acuity (BCVA; ≥0.2 logMAR), and Nd:YAG laser capsulotomy-related SVFs on first postoperative day (QS1 increase ≥ 2, see below).

Exclusion criteria were as follows: consent refusal, previous vitreoretinal surgery, complicated cataract surgery, very low distant BCVA (>0.5 logMAR), visually disabling SVFs before Nd:YAG laser capsulotomy (QS1 and QS2 ≥ 2), axial length (AL) > 26 mm or <22 mm, absence of SVFs after Nd:YAG laser capsulotomy (QS1 increase < 2), dry eye syndrome, anterior segment disease or chronic use of preserved eyedrops, macular disease, glaucoma or intraocular pressure-lowering therapy, systemic autoimmune disease, thyroid eye disease or rosacea, and body mass index (BMI) < 18.5 kg/m^2^ or >30 kg/m^2^. Patients were assessed before Nd:YAG laser capsulotomy (baseline), on the first day after capsulotomy (Day 1), and at the final visit (Month 2). Each patient contributed only one eye to the study, and all evaluations and analyses were conducted on a per-patient basis to avoid intra-subject correlation.

A comprehensive ophthalmic examination was performed, including distance BCVA by an Early Treatment Diabetic Retinopathy Study-like chart at 3 m, contrast sensitivity (CS) by a Pelli–Robson-like chart at 1 m (both Vision Chart, CSO, Florence, Italy), anterior segment biomicroscopy with PCO grading [6,18], and mydriatic fundus examination.

At each visit, all patients were requested to complete a non-standardized self-administered questionnaire consisting of three items rated on a 0–4 scale. These items assessed floaters perception (QS1), floaters’ interference with daily activities (QS2), and foreign body sensation, i.e., ocular discomfort (QS3).

Adherence to the dietary intervention was monitored through daily self-reported diaries provided to all participants. All patients reported full compliance (100%) with the 2-month supplement regimen. During the initial anamnesis, all patients declared adherence to a Mediterranean diet, and no specific dietary modifications were recommended during the study period.

**Figure 2:**
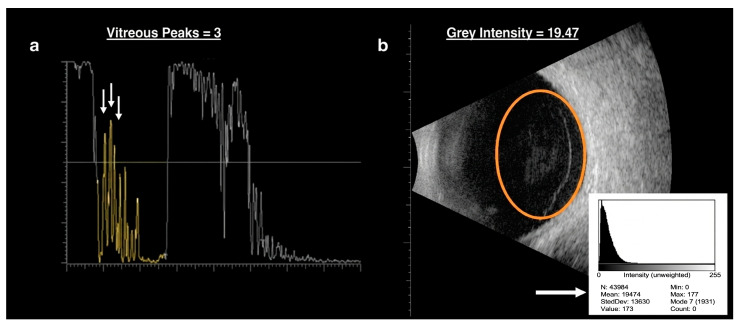
Methods of A-scan (**a**) and B-scan (**b**) analysis. (**a**) Example of an A-scan ultrasound image showing vitreous echoes. Significant vitreous peaks (white arrow) were defined as those exceeding 50% of the vertical axis and were manually counted. (**b**) Example of a B-scan image with superimposed ellipsoidal Region of Interest (ROI, 200 × 280 pixels, orange circle) placed in the posterior vitreous cavity. Gain settings were fixed at 110 dB for all B-scans.

Quantification of floaters was obtained by ocular ultrasound with the AVISO equipment (Quantel Medical, Clermont-Ferrand, France). Two methods are described herein. In the A-scan mode the number of significant vitreous peaks, defined as those with a height exceeding 50%, was first determined and then averaged across four primary longitudinal scans (mean vitreous peaks, MVP), with the average height of the entire scan (AVG Height) arbitrarily set to 70% to enhance vitreous echoes (Figure 2). The ultrasound probe was positioned at the limbus at 3, 6, 9, and 12 o’clock, directed towards the posterior pole, intentionally excluding the axial longitudinal scan to mitigate any artefacts stemming from the intraocular lens.

In the B-scan mode, the intensity of greys, measured on a 0–255 scale, was determined within an ellipsoidal window of fixed dimensions (200 × 280 pixels) manually positioned in the posterior vitreous cavity using the image processing software ImageJ (Software z project tool, version 1.54g, National Institute of Health, Bethesda, MD, USA) and then averaged across one axial sagittal and four primary transversal scans (mean grey intensity, MGI), with the maximal gain of each scan set at 110 dB (Figure 2).

To avoid any influences of the treatment during all of the evaluations, the best effort was made to keep the examinator masked to the group assigned to each participant.

All procedures were conducted under topical anesthesia (oxybuprocaine 0.4%) and pharmacological mydriasis (tropicamide 0.5% plus phenylephrine 10%) by the same experienced operator (AM), using the Q-switched Nd:YAG laser Optimis Fusion (Quantel Medical, Clermont-Ferrand, France) with a capsulotomy contact lens (magnification of ×0.64), and employed the circular technique while aiming to maintain a 4 mm diameter and energy of single spots in the range of 0.5–1.5 mJ.

Only patients complaining of SVFs on the first day following laser capsulotomy (QS1 increase ≥ 2) were selected and then randomly allocated in a 1:1 ratio to the two study groups (control and dietary intervention) until each group reached 20 eyes. No post-randomization exclusions were conducted and the groups remained balanced at Day 1.

Patients assigned to the control group adhered to the standard therapy following Nd:YAG laser capsulotomy, which involved bromfenac 0.09% drops two times a day for 15 days, along with cross-linked hyaluronic acid 0.2% non-preserved drops four times per day for 1 month.

Patients assigned to the dietary intervention received, in addition to the standard therapy, a commercially available oral supplement comprising curcumin, bromelain, and vitreous constituents, with a dosage of 1 tablet per day for 2 months, as recommended by the manufacturer (Table 1).

Randomization was performed using a simple computer-generated sequence (RAND function in Microsoft Excel). The sequence was created and managed by an independent investigator who was not involved in patient enrollment or outcome assessment (GS). Allocation concealment was ensured by using sealed, opaque, sequentially numbered envelopes, which were opened only after confirming participant eligibility.

All assessments, including BCVA, CS, questionnaires, and ultrasonography, were performed by the same experienced examiner (AM) who was masked to group assignments, although not to the timepoint, as visits followed a predetermined clinical timeline.

Baseline parameters were compared between the two groups using Student’s *t* test for continuous variables and the Chi-squared test for categorial ones. The effect of time on each variable and the interaction between the group and time (i.e., the effect of dietary intervention on modifying the progression itself) were assessed using a mixed model for repeated-measures analysis of variance (ANOVA). Group and time served as the between- and the within-subject factors, respectively; the visits on first postoperative day and at 2 months after were specified as the reference and the final, respectively. A total of 40 subjects was determined to attain a statistical power of 0.80 for a mixed ANOVA with two levels for two variables, setting type I error probability at 0.05 and supposing a partial eta-square (η^2^_p_) of 0.05 (moderate effect) for the within–between interaction. Due to multiplicity, the Holm–Bonferroni method was used to control for a family-wise error rate of no more than 0.05, considering a total of seven null hypotheses for the ANOVA within–between interaction terms, one for each outcome variable. Missing data were handled by the pairwise deletion method, according to the default method for managing missing data of the statistical software employed in this analysis; however, the best effort was made to ensure a 2-month visit for each participant. The statistical analysis was performed using SPSS software version 26 (IBM, Armonk, NY, USA) and charts were created using Prism software version 10 (GraphPad, San Diego, CA, USA).

## 3. Results

A total of 239 consecutive eye candidates for laser capsulotomy were screened, 85 of which (36%) resulted to be eligible for the study and, from this cohort, only 40 eyes (47%) exhibiting Nd:YAG-related SVFs on the first postoperative day were included and randomly assigned to the two study groups (Figure 1). The dietary intervention and the control groups demonstrated comparable baseline characteristics in terms of gender, age, time from cataract surgery, PCO score, Nd:YAG energy employed, and capsulotomy diameter (*p* > 0.01 for all variables, Table 2).

All parameters (BCVA, CS, QS1, QS2, QS3, MVP, and MGI) were comparable between the dietary intervention and the control group both at the baseline and the first day after capsulotomy (*p* > 0.01 for all, Table 3).

**Figure 3:**
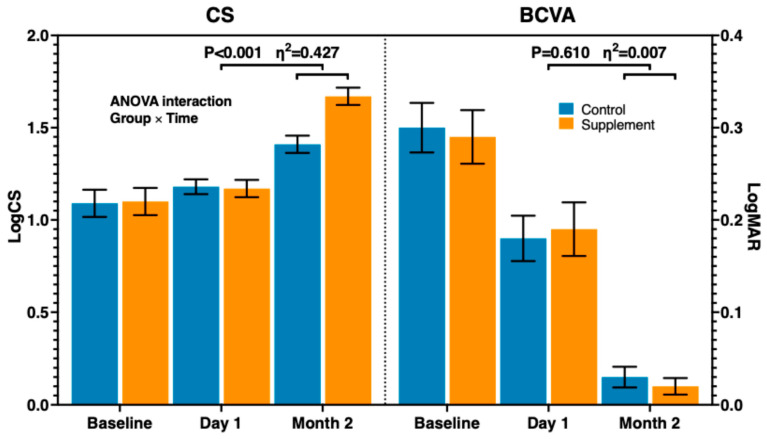
Mean changes in visual function (CS and BCVA); x-axis indicates the visit, y-axis indicates the logarithm of contrast sensitivity (LogCS, **left**) and visual acuity expressed in logMAR (**right**).

All the participants completed the final follow-up visit, and at 2 months, the dietary intervention group showed significantly improved outcomes than the control group for all parameters apart from BCVA, which was comparable between the two groups, as confirmed by the Holm–Bonferroni procedure (Table 3 and Table 4 and Figure 3).

On average, in the group taking the dietary supplement, contrast sensitivity was about two triplets higher, questionnaire scores (floaters perception, interference with daily activities, and foreign body sensation) were each about one point lower, ultrasound vitreous peaks were one point lower, and grey intensity was around 40% lower. Adverse events were assessed during the final visit through a standardized verbal interview; solicited events included dyspepsia, rash, altered taste, and diarrhea/constipation. No supplement-related adverse events were reported.

## 4. Discussion

This study is the first exploring the clinical effects of a commercially available dietary supplement containing a mixture of curcumin, bromelain, glucosamine, chondroitin sulphate, sodium hyaluronate, type II collagen, and vitamin C in managing symptomatic vitreous floaters, in particular, those secondary to Nd:YAG laser capsulotomy. These findings suggest a potential short-term efficacy of dietary intervention in reducing symptomatic vitreous floaters 2 months after Nd:YAG laser capsulotomy. Dietary supplement was associated at 2 months with a more significant improvement compared to the control group in terms of contrast sensitivity, subjective questionnaire scores, and objective ultrasound parameters. Nd:YAG laser capsulotomy is thought to induce vitreous floaters by four primary mechanisms: induction of posterior vitreous detachment (PVD) [4], posterior displacement of opaque capsular remnants into the vitreous cavity, which is syneretic in older subjects [5,6], disruption of the anterior hyaloid fibrillary frame [19], and intraocular inflammation induced by laser energy [7].

Although curcumin has demonstrated biological effects in several ocular disease [14,15], the in vivo effects of curcumin on vitreous floaters have yet to be evaluated. Only in vitro studies have been conducted thus far: one on proliferative diabetic retinopathy (PDR) [20] and two on proliferative vitreoretinopathy (PVR) [21,22]. We suppose that curcumin exerts anti-inflammatory effects on the vitreous body, thereby reducing Nd:YAG laser-induced intraocular inflammation.

Bromelain has a recognized proteolytic function on primary and hematic vitreous floaters instead [16,17].

The in vivo role of dietary supplementation with type II collagen and mucopolysaccharides glucosamine, chondroitin sulphate, sodium hyaluronate, and vitamin C in the management of floaters has not yet been evaluated. We hypothesize a remodelling and restorative role in the fibrillary framework, altered by the natural ageing process (synchysis and syneresis) and Nd:YAG laser-induced intraocular inflammation [7]. While two clinical studies demonstrated the effectiveness of oral supplementation with micronutrients (L-lysine, vitamin C, zinc, Vitis vinifera, and Citrus aurantium extracts) in alleviating visual discomfort associated with vitreous opacities after 6 months [23,24], notably, this formulation did not include collagen or mucopolysaccharides.

Contrast sensitivity represents the functional parameter most sensitive to floaters [25], which are thought to negatively affect the quality of life by diminishing CS [7,26]. In our study, CS before capsulotomy was lower than normal subjects of the same age [27], but consistent with the published data [1], and marginally increased just after capsulotomy. We hypothesize that, while baseline CS reduction was due exclusively to PCO, the first day after the procedure, it depended on combined improvement by PCO removal and worsened with SVF occurrence. We documented ultrasound reduction in vitreous opacities at 2 months, more pronounced in the intervention group, which had a nearly full recovery of CS, in contrast to the control group, which instead was still on average about two triplets below the normal range.

A classic Snellen or ETDRS chart for visual acuity cannot effectively measure floaters [28], as both test a fixed and high-contrast (100%) stimulus within only a few central degrees of the visual field [7]. In our study, BCVA also proved inadequate in defining the functional impact of SVFs, as both groups fully recovered by two months without any discernible difference, despite distinct ultrasound findings and subjective complaints.

Symptomatic vitreous floaters are known to reduce vision-related quality of life [29,30]. In our study, at 2 months, the dietary intervention was associated with a more significant improvement of self-reported floaters perception and their interference with daily activities compared to the controls, which scored on average about one point higher. The advantage of questionnaires is the ability to quantify symptoms experienced by patients. However, the subjectivity of patients is the main drawback, as the discomfort associated with floaters is closely linked to psychological aspects. It is unclear whether floaters exacerbate pre-existing psychological conditions or if subjects with psychological disorders (e.g., depression, anxiety, and stress) overestimate floaters by focusing on that symptom [31].

Multiple ultrasound methods have been suggested to quantify vitreous floaters. Minimum Image Gain (MIG) was employed as a measure of vitreous hemorrhage density [32,33,34,35] and involves reducing the gain from 110 dB to the point where the vitreous cavity becomes completely echo-silent, and vitreous opacities or hemorrhage are no longer visible on a standard temporal longitudinal B-scans. Quantitative Ultrasound (QUS) was used in primary floaters and consists of automatic image processing using software (MATLAB 24.1) and calculations of numerical parameters related to the quantity of vitreous opacities (energy and mean of greys, ROI percentage occupied by floaters only in the whole-central vitreous of longitudinal inferotemporal, and nasal B-scans) [25]. However, none of these methods have been compared with each other, and there is no consensus on which one is better for quantifying floaters or for determining floaters secondary to Nd:YAG laser capsulotomy.

In our study, we introduced two novel ultrasound methods: the direct count of vitreous peaks (MVP) in the case of A-scans and the calculation of mean grey (MGI) for B-scans using image processing software, which is relatively straightforward. However, the accuracy and reproducibility could not be evaluated, as scans were acquired only once and by the same technician. A distinct progression was observed between the A-scans and B-scans. Both variables exhibited comparable changes just after capsulotomy. However, while MGI remained stable at 2 months in the dietary intervention group despite a mean increase of about 56% in the control group, MVP reduced at 2 months in both groups, especially in the dietary intervention group, which was about on average one peak lower. We attribute this disparity in temporal progression to the nature of the measures: MVP, which solely counts the number of significant peaks, is an instantaneous and raw measure that highlights vitreous opacities irrespective of their distribution and density. Conversely, MGI provides a mean measure, capturing the “background noise” and adjusting for the real presence, density, and distribution of floaters.

Unlike MIG and QUS, which either reduce gain until echo-silence or rely on more complex image analysis (e.g., MATLAB-based processing), our ImageJ-based method for calculating mean grey intensity (MGI) is straightforward, reproducible, and easily adaptable in clinical settings. The manual placement of a fixed-size ROI across five scans enables averaged quantification, potentially reducing variability.

This study also proves the efficacy of dietary intervention in reducing ocular discomfort associated with the laser procedure, as the improvement of self-reported foreign body sensation was significantly greater in the dietary intervention compared to the control group. Laser capsulotomy may alter the ocular surface and damage the corneal epithelium, since it involves the use of contact lenses (mechanic abrasion) and topical anesthesia (epithelial toxicity). While this finding suggests a possible benefit of dietary supplementation in reducing ocular discomfort, the mechanism remains unclear. One potential explanation could be that curcumin exerts a synergistic effect with artificial tears in enhancing the ocular surface, consequently reducing floaters perception. Dry eye is associated with lacrimal hyperosmolarity, which consequently leads to the overexpression of inflammatory mediators on the ocular surface, and curcumin exhibits an anti-inflammatory effect on dry eye syndrome, as shown by preclinical studies in animal models [14,36]. At present, there is no direct evidence assessing the impact of the ocular surface (dry eye) on floaters perception. However, contrast sensitivity is diminished in dry eye [26] and has shown improvement following topical treatment with artificial tears [37,38,39,40], and intraocular straylight (disability glare) is increased in dry eye as well as in patients with floaters [41] and strongly correlates with disability-induced by floaters [28]. Therefore, we hypothesize that the enhancement of the ocular surface may contribute to further reducing floaters perception, however this remains speculative. Future studies should include validated ocular surface metrics—such as the Ocular Surface Disease Index (OSDI) or non-invasive tear break-up time (NIBUT) to test whether ocular surface integrity modulates straylight and contributes to floater-related symptoms.

While this study observed clinical improvements associated with dietary supplementation, the proposed mechanisms of action, such as the anti-inflammatory effects of curcumin, proteolytic effects of bromelain, and vitreous matrix remodelling from mucopolysaccharides and type II collagen, remain hypothetical. These mechanisms were not directly investigated in this study and should be tested in future translational or mechanistic studies to establish causality.

The main limitation of this pilot study was the short follow-up period (2 months); longer observation timepoints (e.g., 6–12 months) are necessary to explore the time–effect relationship of dietary intervention on outcomes. Another limitation was the fixed-dose combination of this commercially available supplement: experiments are needed to disclose the dose–effect relationships with any mutual interactions of each component and to determine the optimal content of single elements. Furthermore, concomitant systemic medical therapies were not systematically recorded, which may represent an additional confounding factor. In addition, ultrasound-based methods for floater quantification are inherently operator-dependent. In our study, this aspect was mitigated by employing a single experienced examiner for all measurements. Nevertheless, the lack of automated or standardized ultrasound quantification remains a broader challenge in the field [32,33,34,35], making it difficult to objectively quantify floaters and define the impact of the treatment.

Also, the lack of a placebo in the control group, combined with the subjectivity of floaters perception, might have overestimated the improvement in the supplemented group; however, the best effort was made to mask the examiner to treatment assignments, and both functional (contrast sensitivity) and objective (ultrasound) measurements were included. Future studies should incorporate placebo-controlled designs and focus on the development or adoption of standardized, operator-independent, and automated methods for objectively assessing vitreous floaters and treatment efficacy.

## 5. Conclusions

In this pilot randomized study of patients with day-1 symptomatic vitreous floaters (SVFs) after Nd:YAG capsulotomy, short-term supplementation with a curcumin–bromelain–vitreous constituent formulation was well tolerated and associated with greater improvements in contrast sensitivity, ultrasound-based floater metrics (MVP and MGI), and floater-related symptoms compared with standard care alone, while BCVA remained unchanged. While these findings suggest potential short-term benefits of supplementation, conclusions are limited by the small sample size, short follow-up, absence of a placebo control, and lack of reproducibility testing for ultrasound metrics. Confirmatory, placebo-controlled trials with longer follow-up and standardized, objective floater quantification are warranted to validate efficacy and clarify the underlying mechanisms.

## Figures and Tables

**Table 1:** Composition of one tablet of dietary supplement (Vitreolisina Forte, OFFHealth, Florence, Italy).

Molecule	Dosage
Curcumin	190 mg (*Curcuma longa* L. rhizome dry extract, titration 95% curcumin)
Piperine	2 mg
Bromelain	100 mg (*Ananas comosus* L. Merr. stem dry extract, 2500 GDU/g)
Vitreous constituents	
Glucosamine	50 mg
Chondroitin sulphate	50 mg
Sodium hyaluronate	30 mg
Type II collagen	10 mg
Vitamin C	50 mg

**Table 2:** Demographics and baseline characteristics.

Variable	Control (*n* = 20)	Intervention (*n* = 20)	*p*-Value ^a^
Gender (M/F)	10/10 (50%)	12/8 (60%)	0.751
Age (years)	71.1 (6.1)	69.2 (5.9)	0.325
Cataract surgery (years before)	5.4 (2.4)	5.1 (2.2)	0.682
PCO (score)	2.8 (1.1)	2.7 (1.1)	0.744
BCVA (LogMAR)	0.30 (0.12)	0.29 (0.13)	0.863
CS (LogCS)	1.09 (0.33)	1.10 (0.33)	0.888
Nd:YAG energy (mJ)	47.0 (12.0)	45.9 (13.0)	0.784
Capsulotomy diameter (mm)	4.3 (0.3)	4.4 (0.3)	0.463

Abbreviations: BCVA (best-corrected visual acuity), CS (contrast sensitivity), and PCO (posterior capsular opacity). Note: Data are means (standard deviations) and number (%). ^a^ Intervention vs. control group; Student’s *t* test for continuous and scaled variables; Chi-squared test for categorial variables.

**Table 3:** Summary of visual function, questionnaire scores, and ultrasound parameters follow-up.

Variable	Baseline			Day 1			Month 2		
Group	Control (*n =* 20)	Intervention (*n* = 20)	*p*-Value ^a^	Control (*n* = 20)	Intervention (*n* = 20)	*p*-Value ^b^	Control (*n* = 20)	Intervention (*n* = 20)	*p*-Value ^b^
BCVA (LogMAR)	0.30 (0.12)	0.29 (0.13)	0.802	0.18 (0.11)	0.19 (0.13)	0.796	0.03 (0.05)	0.02 (0.04)	0.478
CS (LogCS)	1.09 (0.33)	1.10 (0.33)	0.888	1.18 (0.18)	1.17 (0.19)	0.900	1.41 (0.21)	1.67 (0.21)	<0.01
QS1 (score) ^c^	0.55 (0.51)	0.60 (0.60)	0.778	3.10 (0.31)	3.00 (0.80)	0.603	2.75 (0.79)	1.65 (0.81)	<0.01
QS2 (score) ^c^	0.30 (0.47)	0.45 (0.51)	0.340	1.85 (0.67)	1.95 (0.69)	0.644	1.75 (0.97)	0.85 (0.59)	<0.01
QS3 (score) ^c^	0.45 (0.51)	0.40 (0.50)	0.757	2.10 (0.55)	2.20 (1.15)	0.728	1.10 (0.91)	0.20 (0.41)	<0.01
MVP (counts) ^c^	0.95 (0.85)	0.85 (0.66)	0.679	5.05 (0.93)	5.15 (1.28)	0.779	3.20 (0.89)	2.10 (0.78)	<0.01
MGI (intensity) ^c^	12.54 (6.12)	13.23 (5.67)	0.714	20.53 (9.41)	20.22 (9.98)	0.921	31.99 (13.28)	19.10 (8.34)	<0.01

Abbreviations: BCVA (best-corrected visual acuity), CS (contrast sensitivity), MGI (mean grey intensity), MVP (mean vitreous peaks), QS1 (floater perception), QS2 (interference with daily activities), and QS3 (foreign body sensation). Note: Data are mean (standard deviation). ^a^ Intervention vs. control group (Student’s *t* test for independent samples). ^b^ Intervention vs. control group (Bonferroni-adjusted ANOVA pairwise comparisons). ^c^ QS1–3 are on a 0–4 scale each. MVP is the mean counts of A-scan vitreous peaks > 50% height. MGI is the mean intensity of B-scan grey on a 0–255 scale.

**Table 4:** ANOVA Interaction group × time at follow-up.

Variable	Statistic (*F*) ^b^	Significance (*p*) ^c^	Effect size (η^2^_p_) CI 95% ^a^
BCVA	0.264	0.610	0.007 (<0.001–0.141)
CS	28.303	<0.001	0.427 (0.181–0.636)
QS1	13.058	0.001	0.256 (0.052–0.491)
QS2	16.102	<0.001	0.298 (0.077–0.529)
QS3	7.917	0.008	0.172 (0.013–0.407)
MVP	24.732	<0.001	0.394 (0.151–0.610)
MGI	24.549	<0.001	0.392 (0.150–0.609)

*Abbreviations*: BCVA (best-corrected visual acuity), CS (contrast sensitivity), MGI (mean grey intensity), MVP (mean vitreous peaks), QS1 (floater perception), QS2 (interference of floaters with daily activities), and QS3 (foreign body sensation). *Note*: ^a^ Day 1 after capsulotomy was assumed as the reference visit, and Month 2 as the final visit. ^b^ Statistic (*F*): degree of freedoms (*df*) were 1 and 38 for the interaction and error term, respectively. ^c^ Statistical significance (*p*): it evaluates the interaction between group and time progression of the variable considered. d Partial eta-square (η^2^_p_) with 95% confidence intervals: it evaluates the interaction between group and time progression of the variable considered.

## Data Availability

The original contributions presented in this study are included in the article. Further inquiries can be directed to the corresponding author.

## Abbreviations

The following abbreviations are used in this manuscript:

AL	Axial length
ANOVA	Analysis of variance
AVG	Average height
BCVA	Best-corrected visual acuity
BMI	Body mass index
CS	Contrast sensitivity
ETDRS	Early Treatment Diabetic Retinopathy Study
LogMAR	Logarithm of the minimum angle of resolution
MGI	Mean grey intensity
MVP	Mean vitreous peaks
Nd:YAG	Neodymium-doped yttrium aluminum garnet
NF-κB	Nuclear factor kappa-light-chain-enhancer of activated B cells
PCO	Posterior capsular opacity
PDR	Proliferative diabetic retinopathy
PVD	Posterior vitreous detachment
PVR	Proliferative vitreoretinopathy
QS1	Questionnaire score 1 (floaters perception)
QS2	Questionnaire score 2 (daily activities interference)
QS3	Questionnaire score 3 (foreign body sensation)
QUS	Quantitative ultrasound
ROI	Region of interest
SVF	Symptomatic vitreous floater
